# Adaptive Heterosubtypic Immunity to Low Pathogenic Avian Influenza Viruses in Experimentally Infected Mallards

**DOI:** 10.1371/journal.pone.0170335

**Published:** 2017-01-20

**Authors:** Karen M. Segovia, David E. Stallknecht, Darrell R. Kapczynski, Lisa Stabler, Roy D. Berghaus, Alinde Fotjik, Neus Latorre-Margalef, Monique S. França

**Affiliations:** 1 Poultry Diagnostic and Research Center, The University of Georgia, Athens, Georgia, United States of America; 2 Southeastern Cooperative Wildlife Disease Study, Department of Population Health, College of Veterinary Medicine, University of Georgia, Athens, Georgia, United States of America; 3 Southeast Poultry Research Laboratory, Agricultural Research Service, U.S. Department of Agriculture, Athens, Georgia, United States of America; 4 Department of Population Health, College of Veterinary Medicine, University of Georgia, Athens, Georgia, United States of America; St. Jude Children's Research Hospital, UNITED STATES

## Abstract

Mallards are widely recognized as reservoirs for Influenza A viruses (IAV); however, host factors that might prompt seasonality and trends in subtype diversity of IAV such as adaptive heterosubtypic immunity (HSI) are not well understood. To investigate this, we inoculated mallards with a prevailing H3N8 low pathogenic avian influenza virus (LPAIV) subtype in waterfowl to determine if prior infection with this virus would be protective against heterosubtypic infections with the H4N6, H10N7 and H14N5 LPAIV subtypes after one, two and three months, respectively. Also, we investigated the effect of cumulative immunity after sequential inoculation of mallards with these viruses in one-month intervals. Humoral immunity was assessed by microneutralization assays using a subset of representative LPAIV subtypes as antigens. Our results indicate that prior inoculation with the H3N8 virus confers partial protective immunity against subsequent heterosubtypic infections with the robustness of HSI related to the phylogenetic similarity of the HA protein of the strains used. Furthermore, induced HSI was boosted and followed by repeated exposure to more than one LPAIV subtype. Our findings provide further information on the contributions of HSI and its role in the dynamics of IAV subtype diversity in mallards.

## Introduction

Wild aquatic birds from the order Anseriformes and Charadriiformes are the major reservoir of Influenza A viruses (IAV) [1, 2]. Mallard (*Anas platyrhynchos*) is the most common dabbling duck species in North America and Europe and is an important species in the ecology of avian influenza [3, 4]. Seasonal patterns of IAV infections in waterfowl have been described in the Northern Hemisphere (North America, Europe, and Asia) [1, 5–7] where IAV prevalence increases at the end of the summer and peaks in early fall as a result of the congregation of adult and immunologically naïve young birds in breeding grounds prior to Southern migration [2, 3]. Detection rates drop during winters with slight increases during spring migrations [5, 8]. Prevalence of IAV is higher among juvenile ducks as compared to adults, which is probably a result of immunity induced by previous IAV infections in adult birds [5, 9, 10]. In North America, the more common HA subtypes of IAV reported in ducks are H3 and H4, followed by H1, H2, H6, H7, H10, and H11, while the H14 subtype has rarely been detected in surveillance studies [3, 5, 11, 12]. Although the H4 and H6 subtypes were also frequent in surveillance studies in Europe, recurrent detection of other subtypes was not significantly different [5, 13]. Seasonal patterns of IAV prevalence among wild birds have been described; however, factors and mechanisms that drive diversity and prevalence of IAV subtypes such as the effects of homo- and heterosubtypic immunity remain unclear [14, 15]. Previous studies have demonstrated the induction of homosubtypic and partial heterosubtypic immunity in mallards [16–20], and this has been supported further by field observations [21]. At the same time, additional studies are needed to understand better the effect of reinfections with common and less frequently detected subtypes of LPAIV on the ecology of influenza in the wild bird reservoir.

The objectives of this research were to investigate *i)* the protective effect induced by prior infection with H3N8 LPAIV inoculation against subsequent infections with H4N6, H10N7 or H14N5 LPAIV after 1, 2 or 3 months, respectively and *ii)* the cumulative effect of H3N8xH4N6 and H3N8xH4N6xH10N7 infections against subsequent challenges with H10N7 and H14N5, respectively. All HA subtypes used in this study are classified into Group 2 HAs with H3, H4 and H14 subtypes clustering together in the H3 clade and the H10 subtype clustering in the H7 clade [21–23]. The neuraminidase (NA) subtypes N5 and N8 are classified into Group 1 within clade N8; whereas N6 and N7 into Group 2 within the N7 clade [24]. We hypothesized that prior LPAIV infections in mallards would induce cross-protective immunity that would reduce viral shedding of subsequent inoculations with phylogenetically closely related LPAIV subtypes.

## Materials and Methods

### Ethics statement

General care and handling of birds were performed in accordance with the guidelines of the Institutional Animal Care and Use Committee (IACUC), as outlined in the Guide for the care and Use of Agricultural Animals in Agricultural Research and Teaching and under an animal use protocol approved by the IACUC at the University of Georgia (UGA; AUP# A2012 09-019-Y3-A3).

### Animals

Thirty-five one-day-old mallards were purchased from a commercial waterfowl supplier (eFowl Hatchery, Denver, Colorado, USA). Ducks were raised under approved conditions in the Animal Resources building at The University of Georgia until they were a month old. All ducks were observed twice daily for evidence of clinical disease signs. Three days before each inoculation, the ducks were transferred to high-efficiency particulate (HEPA) filter isolators located in an animal biosafety level 2 (ABSL-2) room for acclimatization.

### Viruses

Four wild bird-origin LPAIV A/mallard/MN/Sg-000169/2007 (H3N8), A/mallard/MN/AI11-4979/2011 (H4N6), A/mallard/MN/AI11-4412/2011 (H10N7) and A/blue winged-teal/TX/AI13-1028/2013(H14N5) were used in this study. Inocula were generated by a second passage of viral stocks in 9- to 11-day old specific pathogen free (SPF) embryonated chicken eggs (ECEs). The viruses were titrated in ECEs, and the 50% embryo infectious dose (EID_50_) was calculated by the Reed and Muench method [25]. Back titrations were performed on the day of the challenge to confirm the titer of inoculated viruses.

### Study design

**Fig 1:**
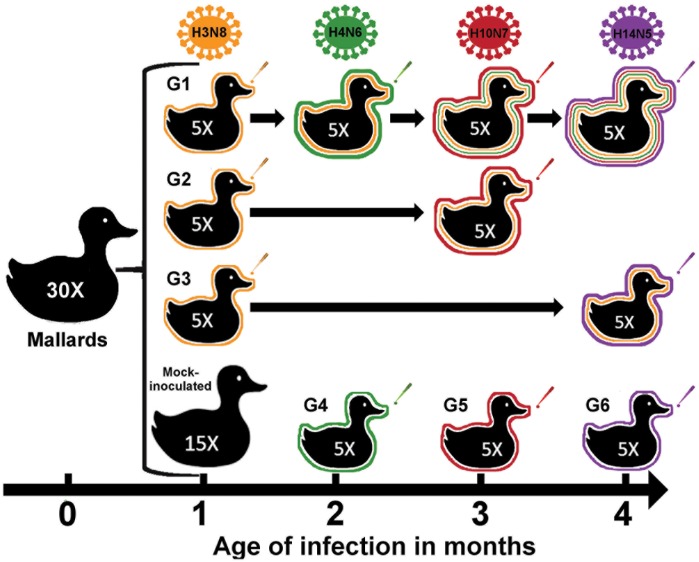
Design of the experimental infections of mallards with different subtypes of LPAIV. Fifteen 1-month-old mallards were inoculated with the H3N8 LPAIV via choanal cleft and randomly divided into three challenge groups with five ducks each. Group 1 was sequentially challenged with H4N6, H10N7 and H14N5 LPAIV at 1-month interval each. Group 2 was challenged with H10N7 two months after H3N8 inoculation, while group 3 was challenged with H14N5 after three months. Single infection and age-matched control ducks were included for all inoculations (Fig 1).

Two groups of fifteen one-month-old mallards were inoculated with 10^6^ EID_50_/0.1 ml of the H3N8 virus or the same volume of mock inoculum via the choanal cleft, respectively. One month later, at two months of age, birds were divided into six groups of five ducks each. Groups 1, 2 and 3 (G1, G2, and G3) consisted of birds previously inoculated with H3N8 virus, while groups 4, 5 and 6 (G4, G5, and G6) consisted of mock-inoculated ducks and served as naïve birds in following challenges. One month after H3N8 inoculation, G1 and the naïve ducks in G4 were challenged with 10^6^ EID_50_/0.1 ml of the H4N6 virus. Two months after H3N8 inoculation, G1, G2 and the naïve ducks in G5 were challenged with 10^6^ EID_50_/0.1 ml of H10N7. Finally, G1, G3 and the naïve ducks in G6 were challenged with 10^6^ EID_50_/0.1 ml of H14N5, three months after H3N8 inoculation. The experimental design is schematically shown in Fig 1.

After completing the experiments in groups G4 and G5, birds were euthanized with CO_2_ and cervical dislocation at 3 and 4 months of age respectively. All remaining ducks, which tested negative for IAV at five months of age by virus isolation and RT-PCR, were subsequently transferred to the Southeast Poultry Research Laboratory, U.S. National Poultry Research Center, Agricultural Research Service, U.S. Department of Agriculture, Athens, GA, USA for a follow-up study.

Oropharyngeal (OP) and cloacal (CL) swabs were collected once daily on days 0–8, then on 10, 12 and 14 days post-inoculation (dpi). Swabs were placed into 2 ml of sterile brain-heart-infusion (BHI) supplemented with antibiotics (penicillin 1 000 units/ml, streptomycin 1 mg/ml, amphotericin B 25 μg/ml, gentamycin 250 μg/ml and kanamycin 500 μg/ml). Individual blood samples were obtained from the brachial or jugular vein on 0 and 14 dpi and processed 1–2 hours after sampling; tubes were centrifuged at 1500 *g* for 10 min, and sera was separated and stored at -20 C until testing.

### Virus isolation

Virus isolation from OP and CL swabs was attempted through inoculation of 9- to 11- day-old SPF chicken embryos as previously described [25]. Allantoic fluid from each inoculated egg was screened for virus by hemagglutination (HA) assay using 0.5% chicken red blood cells [25].

### RNA extraction and quantitative real-time reverse transcriptase polymerase chain reaction (qRT-PCR)

Viral RNA extraction was carried out from 50 μl of the OP and CL swab supernatants with the MagMAX-96 AI-ND viral RNA isolation kit (Ambion, Austin, TX, USA) using the automated KingFisher^™^ magnetic particle processor (Thermo Fisher Scientific, Pittsburgh, PA, USA) according to the manufacturer’s specifications slightly modified [26]. RNA was eluted with 50 μl of elution buffer and stored at -80°C until testing.

Previously described primers and probe targeting the Influenza A virus matrix gene [27] were used to perform the qRT-PCR reaction by using the One-Step RT-PCR Kit (QIAGEN, Valencia, CA, USA) on a StepOne Real-time PCR System (Applied Biosystems, Darmstadt, Germany). Briefly, one μl of kit-supplied enzyme mixture, 0.4 μM of each primer, 0.12 μM probe, 320 μM of each dNTP, 3.75 mM MgCl_2_ and 13 U of RNase inhibitor (Promega, Madison, WI, USA) were used in a 25 μl reaction. Cycling conditions consisted of reverse transcription for 30 mins at 50°C and denaturation for 15 mins at 94°C, followed by 45 cycles of 0 s at 95°C and 1 min at 60°C. Samples with Ct values ≤ 40 were considered positive for Influenza A virus. We arbitrarily assigned a Ct value of 45 to samples showing undetermined RT-PCR results for statistical analyses. All titrated viral stocks were serially diluted for RNA extraction and RT-PCR. Standard curves were generated to convert the experimental Ct values to viral titers in EID_50_/ml equivalents.

### Subtype-specific RT-PCR

To confirm that ducks were effectively infected and shedding the virus inoculated at each time point, subtype-specific RT-PCRs were performed on the viruses isolated from OP and CL swab samples collected on day three post-inoculation. Previously described primers for H3 [28], H4 [29], H10 [30] and H14 [31] were used for subtype identification.

### Serology

All serum samples were analyzed using the AI MultiS-Screen Ab ELISA kit (Idexx, Westbrook, ME, USA) according to the manufacturer's protocol. Samples with signal-to-noise ratio values ≤0.5 were considered positive to Influenza A virus nucleoprotein (NP). Sera also were tested by microneutralization (MN) as previously described [32]. Viruses used as antigens for MN assays included A/mallard/NJ/AI10-4263/2010 (H1N1), A/mallard/MN/AI08-2755/2008 (H2N3), A/mallard/MN/AI10-2593/2010 (H3N8), A/mallard/MN/AI10-3208/2010 (H4N6), A/mallard/MN/AI11-3933/2011 (H5N1), A/mallard/MN/SG-01048/2008 (H6N1), A/mallard/MN/AI09-3770/2009 (H7N9), A/mallard/MN/AI08 2721/2008 (H8N4), A/RUTU/NJ/AI07-293/2007 (H9N1), A/RUTU/DE/AI11-809/2011 (H9N2), A/mallard/MN/SG-00999/2008 (H10N7), A/mallard/MN/SG-00930/2008 (H11N9), A/mallard/MN/SG-3285/2007 (H12N5), A/blue-winged teal/TX/AI13-1028/2013 (H14N5), and A/wedge-tailed shearwater/Western Australia/2327/1983 (H15N6). Antibody titers were converted to log_2_ for all analyses.

### Statistical analysis

Comparisons of the duration of viral excretion and neutralizing antibody titers between groups were performed by using non-parametric tests (Kruskal-Wallis or Mann-Whitney U). Ct values between groups over time were compared using linear mixed models with bird as a random effect. Post hoc multiple pairwise comparisons were conducted using the Bonferroni adjustment to limit the type I error rate to 5%. Hypothesis test assumed a two-sided alternative hypothesis, and *P* < 0.050 was considered statistically significant. All statistical tests were conducted using a commercially available software (Stata version 14.0, StataCorp LP, College Station, TX) and graphs were generated using GraphPad Prism software version 6.0 (GraphPad Software Inc., San Diego, CA, USA).

## Results

Overt clinical signs were not observed in any of the experimental groups, and all OP and CL samples collected immediately before each inoculation tested negative for IAV by virus isolation and qRT-PCR. Serum samples from all ducks collected before IAV inoculation tested negative by both ELISA and MN. Back titration of the inocula determined a titer of 10^6.2^ EID_50_/0.1 ml for H3N8, 10^6.0^ EID_50_/0.1 ml for H4N6, 10^6.3^ EID_50_/0.1 ml for H10N7 and 10^5.7^ EID_50_/0.1 ml for H14N5. Also, subtyping by RT-PCR of the viruses isolated after each infection confirmed that birds were excreting only the subtype of virus inoculated at each time point (data not shown).

### Virus isolation and qRT-PCR

#### Primary H3N8 inoculation in 1-month-old ducks

**Fig 2:**
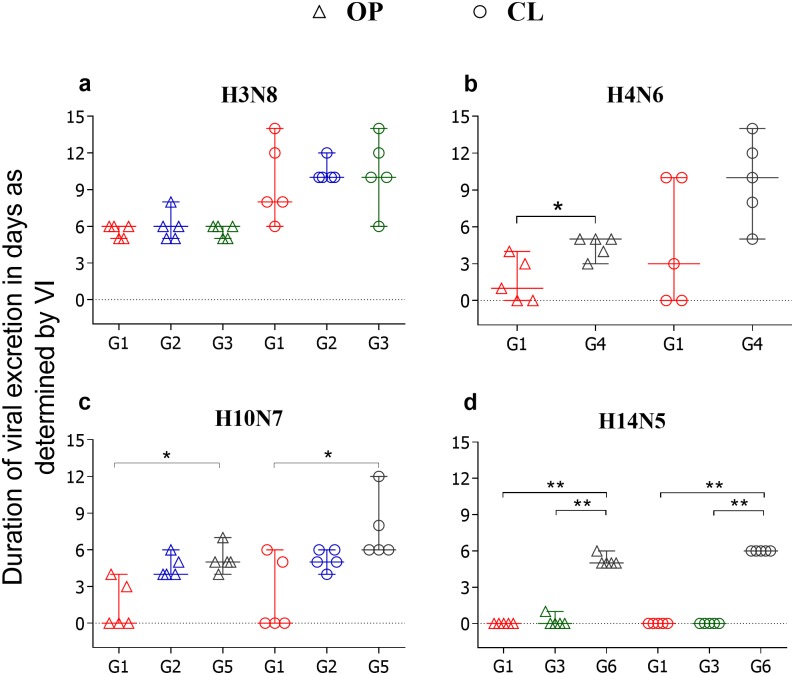
Duration of viral shedding after LPAIV inoculation. Graphs compare duration of virus shedding (median and range) after LPAIV inoculation as demonstrated by VI in oropharyngeal (OP) and cloacal (CL) swabs a) H3N8 inoculation in 1-month-old naïve birds (G1, G2 and G3); b) H4N6 inoculation in 2-month-old birds (G1: H3N8 primed and G4: mock-inoculated) c) H10N7 inoculation in 3-month-old birds (G1: H3N8XH4N6XH10N7-, G2:H3N8 primed, G5: mock-inoculated) d) H14N5 inoculation (G1: H3N8XH4N6XH10N7-, G3:H3N8 primed and G6: mock-inoculated). * and ** denotes significant difference *P* < 0.05 and *P* < 0.01, respectively.

**Fig 3:**
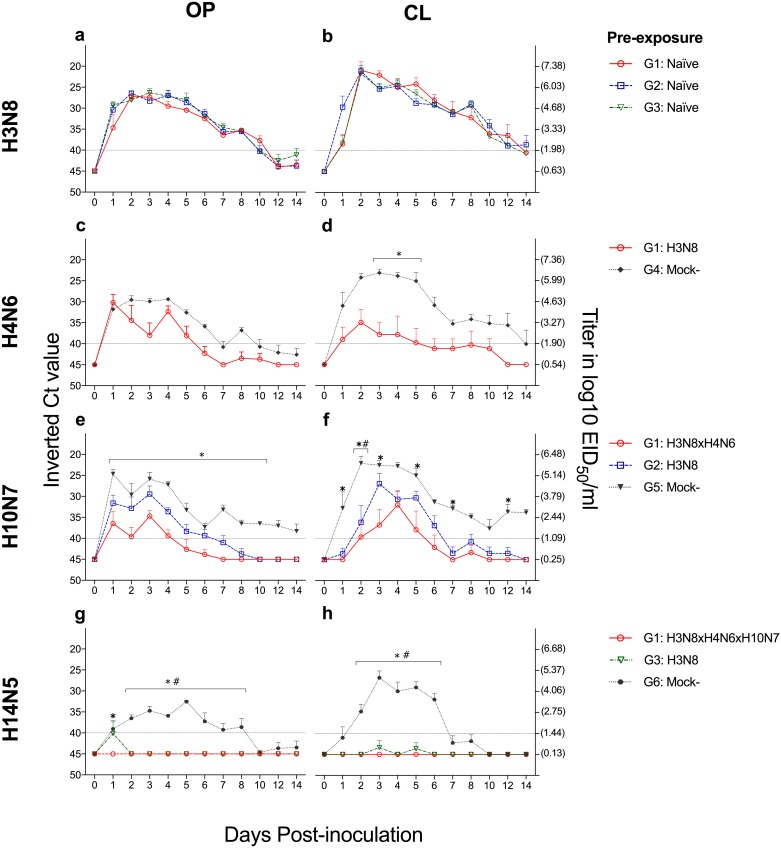
Ct values of RNA viral shedding after LPAIV inoculation. Graphs compare the mean Ct values detected over 14 days in oropharyngeal (OP, left) and cloacal (CL, right) swabs after a,b) H3N8 c,d) H4N6 e,f) H10N7 and g,h) H14N5 inoculation. * denotes significant differences (*P* < 0.05) between G1 and naïve birds after infection with the respective LPAIV, while # between G2 or G3 with the same virus.

Fifteen one-month-old mallards were inoculated with the H3N8 virus to investigate whether this virus would induce cross-protective immunity against heterosubtypic infections. Viral shedding started at 1 dpi in OP and CL swabs, and the duration of viral excretion of H3N8 was not significantly different among groups (G1, G2 and G3), for OP (*P >* 0.999) or CL (*P =* 0.853) swabs (Fig 2a). The peak of H3N8 viral RNA excretion detected by qRT-PCR was at 2 and 3 dpi for OP swabs and 2 dpi for CL swabs. Intermittent viral RNA shedding continued for 8 dpi in all birds, with a general range of 8 to 14 days in OP swabs and 10 to 14 days in CL swabs. The Ct values for OP (*P =* 0.065) and CL (*P =* 0.673) swabs did not show significant differences between groups (G1, G2 and G3) over time (Fig 3a and 3b). All mock-inoculated ducks (G4, G5, G6) tested negative for IAV by virus isolation and RT-PCR (data not shown).

#### H4N6 inoculation in 2-month-old ducks

To investigate whether the previous infection of ducks with H3N8 would confer heterosubtypic immunity against H4N6, 2-month-old ducks from the groups G1 (H3N8 primed) and G4 (mock-inoculated) were challenged with the H4N6 virus. The results showed statistically significant differences in the duration of viral excretion between groups for OP (P = 0.040) but not for CL (P = 0.127) swabs (Fig 2b). However, significant differences in Ct values between groups at 3, 4 and 5 dpi in CL swabs (Fig 3d) but not in OP swabs at any time point were observed (Fig 3c). Overall, we observed a reduction in the duration of the excretion of viable virus and viral RNA after challenge with the H4N6 in the group previously inoculated with the H3N8 virus, correlated with the induction of partial level of cross-protective immunity.

#### H10N7 inoculation in 3-month-old ducks

To investigate whether previous infection with H3N8 or H3N8xH4N6 induced cross-protective immunity against H10N7, the experimental groups G1 (H3N8xH4N6 primed), G2 (H3N8 primed) and G5 (mock-inoculated) were challenged with the H10N7 virus when ducks were 3-months-old. All ducks in G5, which served as the control group in the infection with this virus, started viral shedding at 1 dpi in both OP and CL swabs. In G2, OP and CL viral shedding was delayed by 1 or 2 days, respectively. In G1, viral excretion was delayed by 1 day in 1/5 birds and not detected by virus isolation in 3/5 birds throughout the study period. Statistically significant differences in duration of OP (*P =* 0.012) and CL (*P =* 0.016) viral shedding were detected only among groups G1 and G5 (Fig 2c). There were statistically significant differences in the amount of viral RNA excretion between groups G1 and G5 from 1 to 10 dpi in OP swabs, and at 1, 2, 3, 5, 7 and 12 dpi in CL swabs (Fig 3e and 3f). Hence, these results show that previous infection of ducks with two LPAIV subtypes (H3N8XH4N6), confers a stronger cross-protective immunity against H10N7 as compared to previous infection with only one virus subtype (H3N8).

#### H14N5 inoculation in 4-month-old ducks

To investigate whether previous infection with H3N8 or H3N8XH4N6XH10N7 viruses induced cross-protective immunity against H14N5 virus, the experimental groups G1 (H3N8xH4N6xH10N7 primed), G3 (H3N8 primed) and G6 (mock-inoculated) were challenged with the H14N5 virus when ducks were 4 months old. Ducks from the G6 group started OP and CL viral shedding at 1 dpi, the OP swab of only one bird in G3 tested positive at 1 dpi, and CL viral excretion was not detected by virus isolation in this group. OP and CL viral shedding were completely abrogated in G1, which was demonstrated by virus isolation and qRT-PCR results. There were statistically significant differences in the duration of viral excretion between groups G1 and G6 (*P* = 0.004) and between groups G3 and G6 (*P* = 0.014) (Fig 2d). No statistically significant differences in the duration of viral shedding were found between groups G1 and G3 (*P* = 1.00) (Fig 2d). Ct values in groups G1 and G3 were significantly higher than G6 from 2 to 8 dpi in OP swabs, and from 2 to 6 dpi in CL swabs (Fig 3g and 3h). These results show that previous inoculation with one (H3N8) or three AIV subtypes (H3N8XH4N6XH10N7) confers a strong cross-protective immunity against H14N5 virus infection in ducks.

### Serology

A blocking ELISA assay was performed to confirm seroconversion after inoculation with each IAV used in this study. All ducks inoculated with IAV showed seroconversion at 14 days post-challenges (data not shown).

#### Microneutralization (MN) assay

MN assays were performed using a panel of different subtypes of IAV as antigens to determine if serum samples collected at 14 dpi contained homo- or heterosubtypic neutralizing antibodies. The MN assay was used because of its higher sensitivity as compared with hemagglutination inhibition test [33].

Post H3N8 inoculation: Fourteen out of fifteen birds tested positive for the homologous H3N8 virus on 14 dpi. Also, priming with H3N8 induced cross-reactive antibodies against H14N5 subtype in 2/15 serum samples (log_2_ 4.32, 4.32). Cross-reactive antibodies against the other IAV subtypes tested were not detected (Table 1).

**Table 1:** Microneutralization titers against homo- and heterosubtypic LPAIV in mallards. The table shows microneutralization (MN) titers expressed in log_2_ (median and range) detected in samples collected at 14 days post inoculation with H3N8, H4N6, H10N7 and H14N5 LPAIV and at 1, 2, 3 and 4 months of age, respectively.

Age	Group	Primed with / Inoculum	Group 1								Group 2			
			H3 Clade						H7 Clade		H9 Clade			
			H3N8		H4N6		H14N5		H10N7		H9N2		H12N5	
			Positive samples	Median log_2_ MN titers (range)	Positive samples	Median log_2_ MN titers (range)	Positive samples	Median log_2_ MN titers (range)	Positive samples	Median log_2_ MN titers (range)	Positive samples	Median log_2_ MN titers (range)	Positive samples	Median log_2_ MN titers (range)
1 month	G4,G5,G6	Naïvex**Mock-**	0/15	0	0/15	0	0/15	0	0/15	0	0/15	0	0/15	0
	G1,G2,G3	Naïvex**H3N8**	14/15	5.32 (0–8.32)	0/15	0	2/15	0 (0–4.32)	0/15	0	0/15	0	0/15	0
2 months	G1	H3N8x**H4N6**	5/5	8.32 (6.32–8.32)	4/5	6.32 (0–8.32)	1/5	0 (0–5.32)	0/5	0	0/5	0	0/5	0
	G4	Mockx**H4N6**	0/5	0	3/5	4.32 (0–7.32)	2/5	0 (0–4.32)	0/5	0	0/5	0	0/5	0
3 months	G1	H3N8xH4N6x**H10N7**	5/5	7.32 (6.32–8.32)	5/5	5.32 (4.32–7.32)	2/5	0 (0–5.32)	5/5	4.32 (4.32–6.32)	3/5	4.32 (0–4.32)	0/5	0
	G2	H3N8x**H10N7**	5/5	6.32 (4.32–7.32)	0/5	0	1/5	0 (0–4.32)	4/5	7.32 (7.32–8.32)	0/5	0	1/5	0 (0–4.32)
	G5	Mockx**H10N7**	0/5	0	0/5	0	0/5	0	5/5	6.32 (6.32–8.32)	0/5	0	1/5	0 (0–4.32)
4 months	G1	H3N8xH4N6xH10N7x**H14N5**	5/5	7.32 (6.32–8.32)	5/5	6.32 (5.32–8.32)	3/5	5.32 (0–6.32)	4/5	6.32 (0–7.32)	0/5	0	0/5	0
	G3	H3N8x**H14N5**	5/5	7.32 (6.32–8.32)	2/5	0 (0–6.32)	4/5	6.32 (0–8.32)	0/5	0	0/5	0	0/5	0
	G6	Mockx**H14N5**	0/5	0	0/5	0	5/5	8.32 (7.32–8.32)	0/5	0	0/5	0	0/5	0

Post H4N6 inoculation: Four out of five birds in the groups G1 (H3N8xH4N6) and 3/5 in G4 (H4N6) tested positive for the homologous H4N6 virus. Antibodies to H14N5 were detected in 1/5 serum samples (log_2_ 5.32) from the G1 and 2/5 (log_2_ 4.32, 4.32) samples from the G4 group. Cross-reactive antibodies against the other subtypes of IAV tested were not detected after H4N6 inoculation (Table 1).

Post H10N7 inoculation: All birds from G1 (H3N8xH4N6xH10N7), G5 (H10N7) and 4/5 from the G2 (H3N8xH10N7) groups tested positive for the homologous H10N7 virus. Interestingly, 3/5 birds from the G1 group had cross-reactive antibodies against an H9N2 virus (log_2_ 4.32, 4.32, 4.32). Furthermore, 1/5 serum samples from each G2 and G5 groups had cross-reactive antibodies against an H12N5 (log_2_ 4.32) virus (Table 1).

Post H14N5 inoculation: Three out of five serum samples from G1 (H3N8xH4N6xH10N7xH14N5), 4/5 from G3 (H3N8xH14N5) and 5/5 from G5 (H14N5) groups tested positive for the homologous H14N5 IAV. Interestingly, 2/5 samples from G3 group had cross-reactive antibodies against H4N6 (log_2_ 5.32, 6.32). Cross-reactive antibodies against the other IAV tested were not detected after H14N5 inoculation (Table 1).

**Fig 4:**
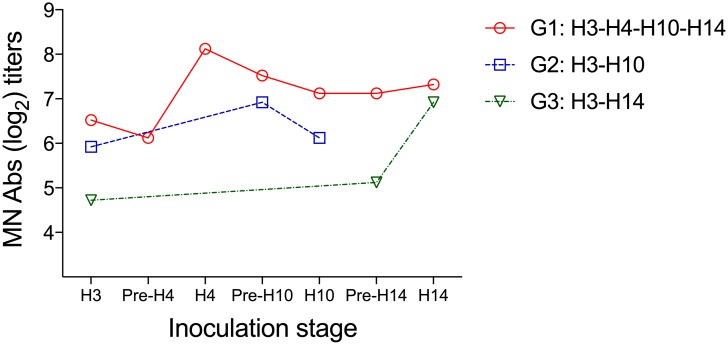
Microneutralization titers against the H3N8 virus after heterosubtypic LPAIV inoculations. The graph compares variation in the mean log_2_ titers against the H3N8 virus in serum samples collected on days 0 and 14 post each heterosubtypic inoculation.

Evaluation of boost in the humoral immune response against H3N8 virus after heterosubtypic challenges: We tested all serum samples from the H3N8 primed birds (G1, G2, and G3) collected prior and after each heterosubtypic inoculation (0 and 14 dpi) by MN assay. We observed an increase in the MN titers against H3N8 virus in the group G1 after H4N6 virus inoculation and in group G3 after H14N5 inoculation. However, no statistically significant differences were detected within groups at any time point (Fig 4).

## Discussion

Previous infection with the H3N8 did not prevent reinfection with the H4N6, H10N7, and H14N5 LPAIVs after one, two and three months, respectively; but induced different levels of protective immunity as evidenced by a decrease in the duration and amount of viral shedding after heterosubtypic infections. The induced protective immunity was boosted following exposure to more than one virus subtype, with complete abrogation of viral shedding upon inoculation with H14N5 in birds previously exposed to three different subtypes (H3N8xH4N6xH10N7). Moreover, three out of five ducks primed with H3N8 were protected against secondary infection with the HA and NA clade-related H14N5 virus three months later, as none of them had detectable levels of viral excretion by virus isolation or RT-PCR after 1 dpi. Shedding of H10N7 was delayed in some birds primed with H3N8 or H3N8xH4N6, and the viral shedding was lower in ducks previously exposed to H3N8xH4N6 viruses.

Based on MN results, there was an induction of cross-reactive antibodies to H14N5 in ducks primed with H3N8, H3N8xH4N6, and H3N8xH4N6xH10N7 viruses; these findings were correlated with a reduction or abrogation in H14N5 virus shedding in groups previously inoculated with H3N8 and with H3N8xH4N6xH10N7 viruses, respectively. Also, infection with H4N6 induced cross-reactive antibodies in some ducks against the HA clade-related H14N5 virus. This finding is in agreement with previous hemagglutination inhibition (HI) results that showed cross-reactive antibodies induced by H4 against H14 subtype but not vice versa [34]. It is generally accepted that virus-specific antibodies neutralize viruses through interaction with the variable regions of the viral HA which prevents its attachment to the host cell receptor; the observed cross-protection may be explained by the affinity of antibodies toward the phylogenetically related HA strains [21]. However, we cannot rule out that antibodies to other major surface proteins such as NA, M2, and NP or other HA epitopes (stem region) may also be playing a role in the cross-protective immunity [35, 36].

Our findings are in agreement with previous experimental studies that reported partial cross-protective immunity conferred by primary H3N8 inoculation against H4N6 virus challenge in mallards [20]. Our data also support results from a longitudinal field study where re-infections with phylogenetically related HA subtypes were rare in mallards, and cross-protection between subtypes within the H1 and H3 clades lasted for at least 30 days [21]. In the present study, we demonstrate that HSI to LPAIV subtypes within the same HA and NA clade can last for at least three months. Seasonal trends of coexistence of IAV and subtype diversity are influenced by the relative host lifespan, population density, and population immunity [1, 14, 37]. The induction of HSI alters the host immune profile and as described here affects the outcome of future infections with IAV in mallards. However, infection with a given IAV in wild birds may induce various levels of protective immunity against subsequent infections with homologous and heterologous subtypes. This variable response could transiently influence the seasonal prevalence of specific viruses [14, 21]. A previous study in Blue-winged Teal (*Anas discors*), an abundant long distance migrant dabbling duck between North America and South America, described H3/H4 and N6/N8 as the most common combination subtypes during the summer and fall [14]. Conversely, H14 and H15 subtypes are absent from most of the surveillance studies, and the former was not reported in North America until 2010 [3, 5, 10, 11]. Hence, our results may add some possible explanations to the cycling patterns in naturally exposed birds, as priming with H3N8 virus induced complete abrogation of H14N5 viral shedding after three months in 3 out of 5 birds.

Although the HI assay is a generally accepted method for assessing the induction of humoral immunity to influenza viruses, we performed the MN assay because of its higher sensitivity, capacity to detect neutralizing antibodies, and its potential to detect cross-neutralizing antibodies [33]. Furthermore, the presence of the truncated form of IgY in ducks, which lacks HI activity, may contribute to neutralizing activity [38]. Additional tests such as HI and neuraminidase inhibition (NI) tests may add further information about the specific cross-reactivity among strains that are phylogenetically related by its HA and/or NA proteins, respectively [34]. None of the serum samples of the G6 (H14N5) tested positive for the HA clade-related H3N8 or H4N6 viruses by MN. It is suggested that these IAV subtypes (H3N8/H4N6), in addition to the H6N2 subtype, are best adapted to wild ducks (mallards, pintails, and Blue-winged Teals) [39]. This fitness might be correlated with a more rapid replication and greater immunogenicity [39, 40] and it is possible that viruses that exhibit greater fitness induce cross-protective immunity against antigenically related and less adapted viruses but, not necessarily the other way [21]. However, additional experimental studies are necessary to test this hypothesis. The detection of low cross-reactive antibody titers to Group 1 IAV (H9N2 and H12N5) in birds in groups G1 (H3N8xH4N6xH10N7) and G5 (H10N7) cannot be explained at this time but may have resulted from NA-related similarities [41]. These observations might also be explained by the existence of broadly neutralizing antibodies targeting the stem region of the HA protein that might confer cross-protective immunity between groups [36].

Previous studies have shown that reinfection with the same virus subtype is capable of boosting homosubtypic immunity as observed in a previous study in mallards inoculated with H3N8 virus [42]. In the present study, we assessed the effect of subsequent heterosubtypic challenges in the induction of neutralizing antibodies against H3N8 virus. We detected an increase in neutralizing antibody titers against the H3N8 virus after challenge with the clade-related HA subtypes H4N6 and H14N5 virus, but there were no statistically significant differences between the groups. We were not able to assess the role of cell-mediated immunity in conferring cross-protective immunity in mallards because lymphocyte proliferation assays used in other species were not replicable in our hands. However, it would be important to determine if cross-reactive, cell-mediated immunity in response to internal proteins as demonstrated for other species is induced when mallards are challenged with different IAV subtypes [43], and if it is influenced by the phylogenetic relatedness of the surface glycoproteins such as HA and NA. Additional studies to assess the role of mucosal immunity in homosubtypic and heterosubtypic infections are also needed, as LPAIV predominantly replicate in the intestinal tract in ducks.

In summary, we showed that single or multiple infections with an LPAIV subtype can induce partial or complete HSI against subsequent challenges in mallards and this cross-protective immunity lasts for at least three months. The robustness and duration of HSI might have important implications in the dynamics of IAVs transmission as well as in the circulation of certain virus subtypes and strains, such as the highly pathogenic H5Nx clade 2.3.4.4 viruses, which were introduced in North America in 2014 by wild birds. The information obtained from this study improves our understanding of the effect of HSI on the ecology of avian influenza in mallards.
